# Hypoglycemia and associated cardiovascular diseases, morbidity and mortality in patients with type 2 diabetes mellitus in university teaching hospitals in Rwanda

**DOI:** 10.3389/jpps.2025.14880

**Published:** 2025-11-27

**Authors:** Jean Baptiste Nyandwi, Pierre Celestin Munezero, Charles Uwambajimana, Gift Crucifix Pender, Jonathan Katandula, Théoneste Umumararungu, Jean Paul Sinumvayo, Ibrahim Eleha Suleiman, Tolessa Muleta Daba, Vedaste Kagisha, Marie Françoise Mukanyangezi, Ahmed Adebowale Adedeji

**Affiliations:** 1 Department of Pharmacology and Toxicology, School of Medicine and Pharmacy, College of Medicine and Health Sciences, University of Rwanda, Kigali, Rwanda; 2 East African Community Regional Centre of Excellence for Vaccines, Immunization and Health Supply Chain Management, University of Rwanda, Kigali, Rwanda; 3 Department of Microbiology and Parasitology, School of Medicine and Pharmacy, College of Medicine and Health Sciences, University of Rwanda, Huye, Rwanda; 4 Department of Pathobiology, Ontario Veterinary College, University of Guelph, Guelph, ON, Canada; 5 Department of Clinical Pharmacy and Pharmacy Practice, School of Medicine and Pharmacy, College of Medicine and Health Sciences, University of Rwanda, Huye, Rwanda; 6 Department of Industrial Pharmacy, School of Medicine and Pharmacy, College of Medicine and Health Sciences, University of Rwanda, Huye, Rwanda; 7 Department of Chemical Pathology and Immunology, University of Ilorin Teaching Hospital, Ilorin, Nigeria; 8 Department of Medical Biochemistry, Molecular Biology and Genetics, School of Medicine and Pharmacy, College of Medicine and Health Sciences, University of Rwanda, Huye, Rwanda; 9 Department of Pharmaceuticals and Biomolecules Analysis, School of Medicine and Pharmacy, College of Medicine and Health Sciences, University of Rwanda, Huye, Rwanda

**Keywords:** hypoglycemia, insulin therapy, type 2 diabetes mellitus, cardiovascular disease, antidiabetic medications

## Abstract

**Background:**

Hypoglycemia is a common yet underrecognized complication in patients with type 2 diabetes mellitus (T2DM), often linked with increased cardiovascular (CV) morbidity and mortality. Despite its clinical importance, there is a limited data on the association between hypoglycemia, CV events, and mortality among T2DM patients in Rwanda. This study investigated the occurrence of hypoglycemia and its association with CV diseases, morbidity, and mortality in T2DM patients attending two university teaching hospitals in Rwanda.

**Methods:**

A retrospective study was conducted using secondary data from 267 T2DM patients attending Kigali University Teaching Hospital (CHUK) and Butare University Teaching Hospital between 2015 and 2020. Socio-demographic and clinical data, including anti-diabetic medications, hypoglycemia episodes, CV events, and comorbidities, were extracted from medical records and analyzed using Python. Binary regression was used to determine significant predictors of hypoglycemia.

**Results:**

Hypoglycemia occurred in 112 (41.9%) patients during their hospitalization or hospital admissions. The use of insulin was significantly associated with hypoglycemia (OR = 1.590, CI: 1.100–2.290, p = 0.010). The mean age of patients who experienced hypoglycemia is 54.2 (±12.1) years. Hypoglycemia occurrence was higher in males (59.8%) group compared to females (40.2%) (p = 0.007). Cardiovascular conditions were common (73.8%), with hypertension being the most prevalent (85.4%). Insulin was the most frequently used anti-diabetic therapy (42.3%). A significant association was found between hypoglycemia and subsequent CV complications. Management of hypoglycemia predominantly involved the use of 50% dextrose solution.

**Conclusion:**

Hypoglycemia is a frequent and clinically significant occurrence among T2DM patients in Rwanda, particularly associated with insulin therapy and CV comorbidities. Enhanced clinical monitoring and individualized treatment regimens are essential to mitigate hypoglycemia-related complications and reduce mortality. It is important to conduct a larger studies to support the evidence based findings and address the current methodological constraints.

## Introduction

Hypoglycemia is a common condition affecting patients with type 2 diabetes mellitus (T2DM) [1]. Patients with hypoglycemia often suffer from symptoms such as dizziness, nausea, physical fatigue, and this is in most cases due to the overdose of oral antidiabetics and injection of insulin therapy [2, 3]. A considerably higher number of patients who develop hypoglycemia are prone to development of cardiovascular (CV) events, with a higher hospitalization rate and are twice more likely to develop stroke compared to non-hypoglycemic ones [4]. Episodes of severe hypoglycemia (SH) in the course of diabetes treatment are accompanied by poor prognosis and frequently lead to mortality [5].

Cardiovascular diseases are a major cause of mortality among patients with T2DM [6]. With reference to the Framingham study on the association between T2DM and CV diseases (CVDs), it was reported that patients with T2DM had a higher incidence of CVDs than those without T2DM [7]. Specifically, the risk of heart disease and stroke is 2–4 times higher in patients with T2DM. Furthermore, it was stated that at least 68% of diabetes patients who are 65 years or older are expected to die as a result of heart disease, while 16% are expected to die of a stroke [8].

The co-occurrence of hypoglycemia and CV events in patients with type 2 diabetes poses more health complications. Certain studies have investigated the relationship between hypoglycemia and CVDs in T2DM patients. In a study conducted in four communities in the United States of America (USA), severe hypoglycemia was found to be associated with coronary heart disease, all-cause mortality, CV mortality, and cancer mortality [9]. However, hypoglycemia was not associated with stroke, heart failure, atrial fibrillation, or non-CV and non-cancer death [9]. Additionally, large clinical trials such as ADVANCE [10], VDAT [11] and Action to Control Cardiovascular Risk in Diabetes ACCORD [12] reported a 3-fold increased risk of hypoglycemic episodes while trying to maintain glycemic goals. Such episodes of hypoglycemia have been reported to be associated with increased incidence of CV events and mortalities [12].

The sustainable development goal (SDG-3), target 3.4, highlights the need to reduce mortality from non-communicable diseases by 2030 [13]. As progress is made towards 2030, the number of people with diabetes are projected to increase by 92% in low income countries and 57% in low-middle income countries [14].

In Rwanda, the prevalence of diabetes mellitus in rural and urban areas is currently 7.5% and 9%, respectively [15]. Evidence has shown that hypoglycemia is common among T2DM patients [1]. Findings from a study that investigated the prevalence and characteristics associated with diabetes mellitus in Rwanda showed that approximately one in six people with diabetes mellitus has hypoglycemia [15]. Increase in age was also reported to be associated with the occurrence of hypoglycemia [15]. However, there is paucity of literature to establish hypoglycemia association with CV events and mortality in Rwanda. Therefore, this study investigated the occurrence of hypoglycemia and its associated CV events and death. It also established the association between anti-diabetic medications, age, sex, and hypoglycemia development in T2DM patients.

## Methodology

### Study design

This study was retrospective and used secondary data collected from medical records of patients with T2DM. The study was conducted between 1 July 2021 and 30 September 2021.

### Study population

The study included files for 267 T2DM patients who attended Internal Medicine or Emergency Departments at Kigali University Teaching Hospital (CHUK) and Butare University Teaching Hospital (CHUB), between 2015 and 2020, were enrolled in the study. These included 141 from CHUK and 126 from CHUB.

### Inclusion and exclusion criteria

Patients with a confirmed diagnosis of T2DM and complete medical records available at CHUK and CHUB between 2015 and 2020 were included. Records were required to provide sufficient information on hypoglycemia (blood glucose level lower than or equal to 70 mg/dL), CV events, and treatment outcomes to allow meaningful analysis. Records that were incomplete, missing critical details, or had unclear diagnoses were excluded to ensure the reliability and accuracy of the findings.

### Data management

A data collection form was used to collect both socio-demographic and clinical data from the participants’ medical files. Electronic entry of the collected data was done for further analysis and interpretation. Clinical data included number of hypoglycemia episodes during hospitalization, CV events such as stroke, myocardial infarction, congestive heart failure, peripheral arterial disease, and hypertension, as well as hospital mortality, comorbidities, and diabetes medications mostly taken by the patients. The information on age, sex, occupation, health insurance type, and marital status was collected for socio-demographic assessment. Datasets were anonymized to protect confidentiality and stored on secure, password-protected computers.

### Statistical analysis

The data collected were analyzed using Python. Analyses were performed using the *pandas*, *numpy*, *statsmodels*, *matplotlib,* and *scikit-learn* packages. Data were summarized by means and standard deviation for continuous variables, while frequencies and percentages were used for categorical variables. Quality checks included standardizing variable entries, verifying plausible ranges, removing duplicate patient records. Only records meeting inclusion criteria were analyzed; any remaining missing values in covariates were handled by case-wise deletion in regression analysis. The proportions of patients with hypoglycemia and CV events, morbidity and all-cause mortality were determined. Binary Logistic Regression (BLR) was used to examine associations between hypoglycemia, CV outcomes, and mortality. Available covariates included age, sex, and clinical variables. Results were reported as odds ratios (ORs) with 95% confidence intervals. A p-value less than 0.05 was considered as statistically significant.

### Ethical consideration and approval

Ethical approval for the study was obtained from the University of Rwanda, College of Medicine and Health Sciences Institutional Review Board and from Ethics Committee at CHUB and CHUK with approval numbers CMHS/IRB/295/2021, REC/UTHB/057/2021, and EC/CHUK/136/2021, respectively. The study was conducted in compliance with the Helsinki’s Declaration of 1964. Patients’ information was coded to protect individual privacy.

## Results

### Demographic and clinical characteristics of participants

In the present study, a total of 267 case records of patients with T2DM and hypoglycemia were examined. The demographic information of the patients is shown in Table 1. The mean age of the patients was 58 years (±12.4). The age distribution showed that the most affected patients were aged between 51 and 70 years (53.1%). Almost an equal distribution was recorded for sex; there were 134 (50.2%) males and 133 (49.8%) females. Almost all patients were married (44.6%) and over 70% were utilizing Community Based Health Insurance (MUSA) compared to 12.4% who were using Rwanda Social Security Board (RSSB) insurance. The most prevalent occupation of the patients was farming (21.7%).

**TABLE 1:** Demographic characteristics of patients (n = 267).

Characteristic		n	Percent (%)
Age group (years)	20–40	30	11.2
	41–50	46	17.2
	51–60	69	25.9
	61–70	73	27.3
	71–80	33	12.4
	81–90	13	4.9
	Not reported	3	1.1
Sex	Female	133	49.8
	Male	134	50.2
Marital status	Married	119	44.6
	Single	17	6.3
	Widowed	28	10.5
	Divorced	9	3.4
	Not reported	94	35.2
Occupation	Teacher	8	3.0
	Farmer	58	21.7
	Retired	20	7.5
	Others	40	15.0
	None	8	3.0
	Not reported	133	49.8
Healthcare insurance	RSSB (RAMA)	33	12.4
	MUSA	191	71.5
	Private insurances	26	9.7
	None	11	4.1
	Not reported	6	2.3

RSSB: rwanda social security board; MUSA: Mutuelle de Sante (Community-based Health Insurance).

Regarding clinical characteristics (Table 2), most of patients (73.8%) had a history of cardiovascular (CV) conditions, and 85.4% had developed hypertension. Other CV conditions were less common, with 8.1% experiencing stroke, while myocardial infarction, congestive heart failure, and peripheral arterial disease each affected less than 1% of participants. Among disease interconnected to CV conditions, renal diseases were present in 20.8% of participants, whereas liver cirrhosis and rheumatoid arthritis were rare, each affecting only 0.5%. Additionally, 36.4% of patients had other comorbidities. Furthermore 79.8% of patients were receiving insulin therapy, indicating that a substantial proportional has potential challenges in maintaining stable blood glucose levels. Additionally, 25.8% developed CV conditions after being diagnosed with T2DM.

**TABLE 2:** Clinical characteristics of participants.

Characteristic	Positive	
	n	(%)
Previous CV condition (n = 267)	197	73.8
Stroke (n = 198)	16	8.1
Hypertension (n = 198)	169	85.4
Myocardial infarction (n = 198)	1	0.5
Congestive heart failure (n = 198)	2	1.0
Peripheral arterial disease (n = 198)	1	0.5
Rheumatoid arthritis (n = 197)	1	0.5
Renal disease (n = 197)	41	20.8
Liver cirrhosis (n = 198)	1	0.5
Insulin (n = 266)	212	79.8
Other comorbidities (n = 198)	72	36.4
CV after (n = 267)	69	25.8

CV: cardiovascular.

### The frequency of hypoglycemia in study patients

**FIGURE 1:**
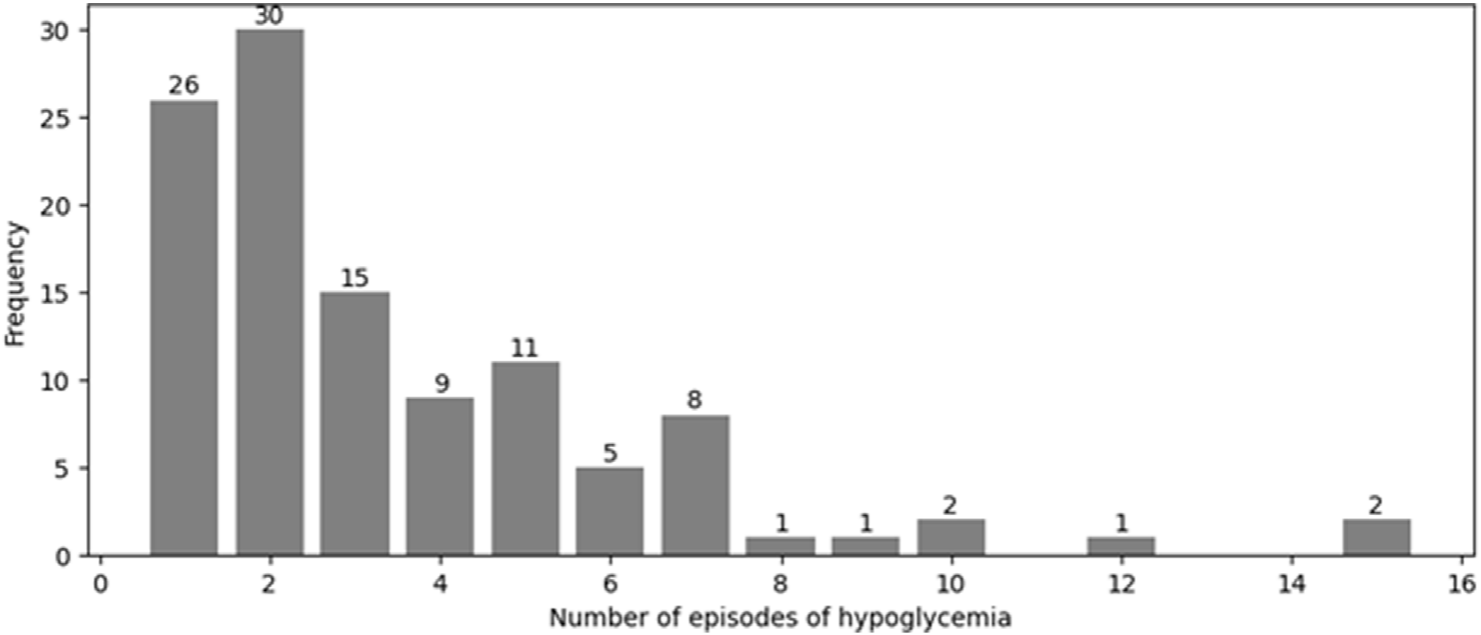
Number of episodes of hypoglycemia occurring among patients with T2DM. Each episode represents a documented event during hospital admissions between 2015 and 2020. Data were extracted retrospectively from medical records, and only events records within this period were included.

This section explores how often hypoglycemic episodes occurred among the patients included in this study. The pattern of hypoglycemia has been characterized in only 112 participants. The highest number of hypoglycemic episodes was 15 and occurred in 2 (1.8%) patients (Figure 1). Most of the patients (30, 27.0%) had two hypoglycemic episodes, followed by 26 (23.4%), 15 (13.5%), and 11 (9.9%) of patients who experienced one, three, and five episodes, respectively.

### Age and sex-based variations in hypoglycemia rates

The proportion of hypoglycemia (Table 3) varied notably across different age groups and between sexes. Among younger individuals aged 20–40 years, hypoglycemia was relatively prevalent, with 56.7% testing positive. In the 40–50 years age group, the proportion was 50.0%. However, as age increased, the proportion of hypoglycemia tended to decrease; in the 51–60 years age group, 43.5% were positive, and this further declined to 37.0% in the 61–70 years group. The lowest proportion was observed in the elder age groups, with only 33.3% of individuals aged 71–80 years and 30.8% of those aged 81–90 years testing positive for hypoglycemia. When considering sex, males had the higher proportion of hypoglycemia at 50.0%, compared to females, who had a proportion of 33.8%.

**TABLE 3:** Distribution of hypoglycemia cases by age group and sex.

Characteristic		Total (n)	Hypoglycemia Positive	
			n	%
Age groups (years)				
	20–40	30	17	56.7
	41–50	46	23	50.0
	51–60	69	30	43.5
	61–70	73	27	37.0
	71–80	33	11	33.3
	81–90	13	4	30.8
Sex				
	Female	133	45	33.8
	Male	134	67	50.0

### Baseline socio-demographic and clinical characteristics of patients by hypoglycemia

The clinical and demographic characteristics of patients with and without hypoglycemia are presented in Table 4. The mean age of patients who experienced hypoglycemia was significantly lower to their non-hypoglycemic counterparts (p = 0.012). The mean age of hypoglycemic and non-hypoglycemic patients was 54.2 (±12.1) and 59.8 (±11.4) years respectively. Males had a higher percentage of hypoglycemia (59.8%) group compared to females (40.2%), a difference that reached statistical significance (p = 0.007). It was revealed that insulin therapy was used in 79.5% hypoglycemic patients and 59.7% non-hypoglycemic patients. Other variables, including hypertension, previous CV conditions, stroke, renal disease, and other comorbidities, showed no significant differences between groups (p > 0.05).

**TABLE 4:** Distribution of demographic and clinical characteristics among T2DM patients with and without hypoglycemia (n = 267).

Characteristics		Hypoglycemia (n = 112)		No hypoglycemia (n = 155)		P-value^a^
Age (mean in years, SD)		54.2	12.1	59.8	11.4	0.012
Age groups (years)		n	%	n	%	0.045
	20–40	17	15.2	13	8.4	
	41–50	23	20.5	23	14.8	
	51–60	30	26.8	39	25.2	
	61–70	27	24.1	46	29.7	
	71–80	11	9.8	22	14.2	
	81–90	4	3.6	9	5.8	
Sex						0.007
	Male	67	59.8	67	43.2	
	Females	45	40.2	88	56.8	
Insulin use		89	79.5	91	59.7	0.010
Hypertension		52	46.4	78	50.3	0.520
Previous CV conditions		18	16.1	23	14.8	0.812
Stroke		10	8.9	6	3.9	0.129
Renal disease		20	17.9	21	13.5	0.139
Other comorbidities		29	25.9	43	27.7	0.730

^a^ P-values were calculated using chi-square or t-test for categorical variables and t-test for continuous variables.

SD: standard deviation; CV: cardiovascular.

### Management approaches provided to study participants

**FIGURE 2:**
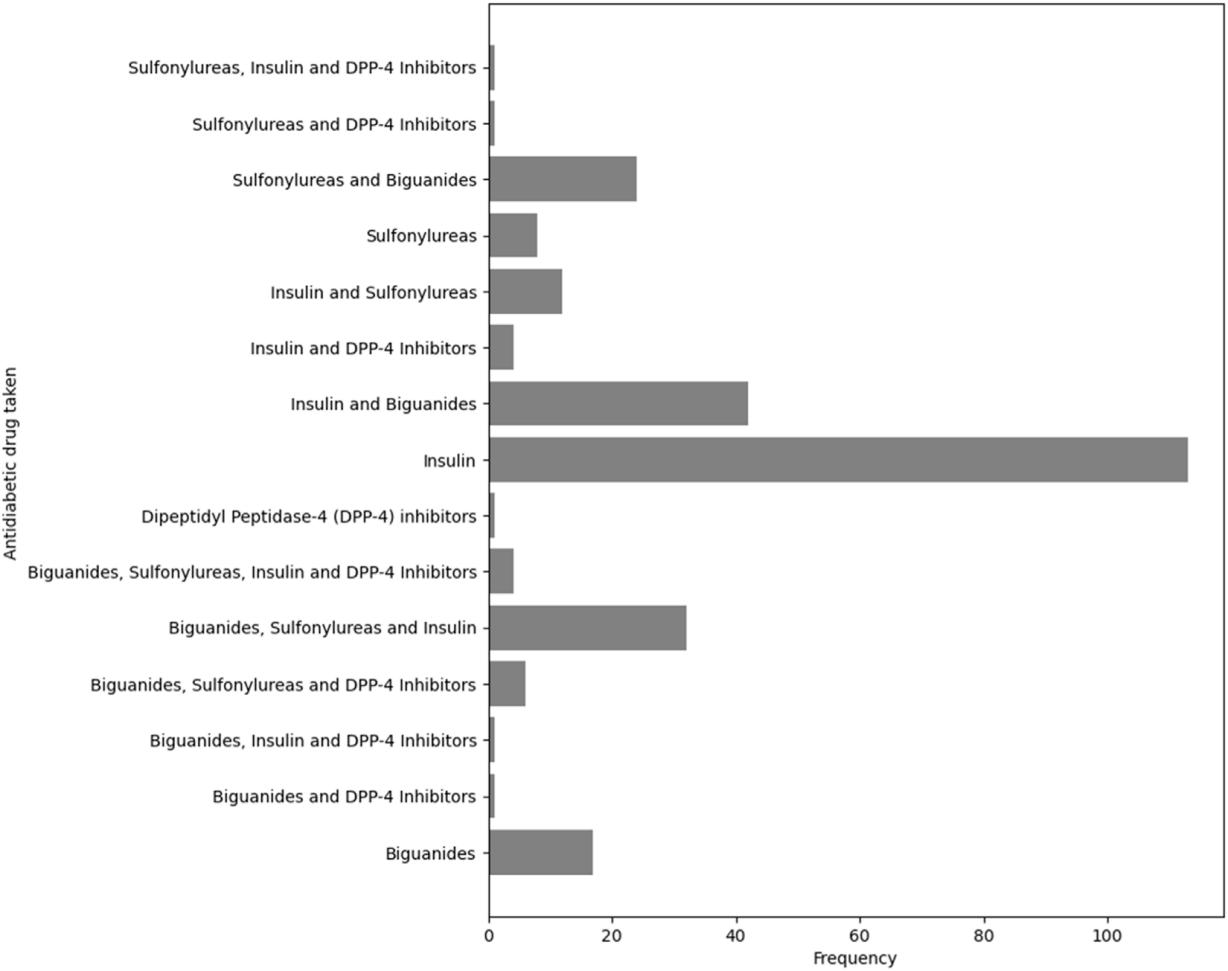
Distribution of anti-diabetic medications among study patients Dipeptidyl Peptidase-4 (DPP-4) inhibitors: DPP-4i).

To understand the management of diabetes among study participants, the types and frequencies of anti-diabetic drugs used were analyzed. The findings indicated that insulin (INS) was the most frequently used medication, with 113 (42.3%) of patients reporting its use (Figure 2). This was followed by the combination of insulin with biguanides, biguanides combined with surfonylureas and insulin, and surfonylureas combined biguanides, used by 42 (15.7%), 32 (12.0%), and 24 (9.0%) of participants, respectively.

The medication administration rates among the study patients were assessed to understand treatment patterns. The findings indicated that while a substantial proportion of the patients received blood pressure-lowering treatments (81.2%), the use of lipid-lowering agents, anti-platelet agents, and others non-specified drugs was less common (16.7%, 16.1%, 14.6, respectively) (Table 5).

**TABLE 5:** Distribution of cardiovascular medications among study patients by drug categories.

Medication	n	Administered	
		Yes	(%)
Blood pressure lowering	197	160.0	81.2
Lipid-lowering agents	197	33.0	16.7
Anti-platelet agents	198	32.0	16.1
Other drugs	198	29.0	14.6

**FIGURE 3:**
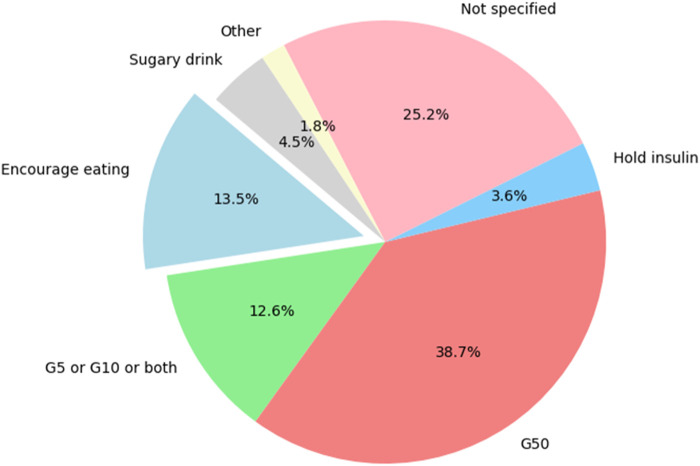
Distribution of various strategies for managing hypoglycemia. (G5: 5% Dextrose in Water; G10: 10% Dextrose in Water; G50: Dextrose in Water).

The results on the hypoglycemia management indicated that the most commonly used strategy was 50% dextrose solution (G50), which accounted for 38.7% of the cases (Figure 3). Not specified methods were noted in 25% of the instances, highlighting a significant portion of patients where the management approach was not explicitly detailed. Encouragement of eating, and the use of 5% or 10% dextrose in water (G5 or G10) or both, were less common, representing 13.5% and 12.6% of the strategies, respectively. Sugary drinks were used in 4.5% of participants, while hold insulin was the least common approach, applied in only 3.6% of instances.

**FIGURE 4:**
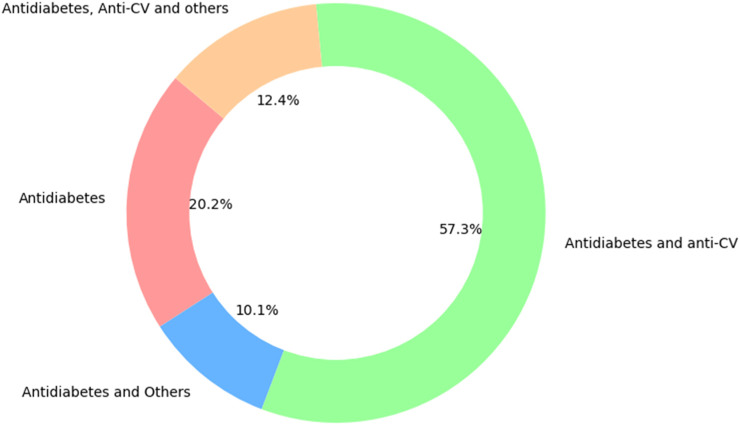
Overall participant management.

Figure 4 illustrates the overall distribution of participant management. Findings revealed that 20.2% of patients were managed with antidiabetes treatment alone. A combined approach of antidiabetes and other treatments was observed in 10.1% of participants. The most common strategy, encompassing both antidiabetes and anti-CV treatments, was employed in 57.3% of participants, highlighting its predominant role in participant management. Additionally, a comprehensive management approach involving antidiabetes, anti-CV and other treatments was utilized in 12.4% of participants.

### Overall participants’ management outcomes

**FIGURE 5:**
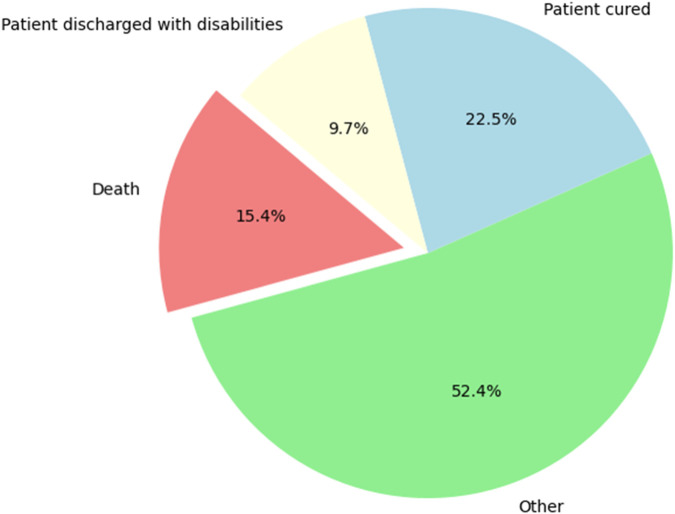
Distribution of overall participant management outcomes, showing the frequency of each outcome category.

The outcomes reported in this study reflect the clinical course of patients admitted with confirmed T2DM who experienced hypoglycemia during hospitalization, either as primary reason for admission or as a documented complication during their hospital stay. A significant proportion of patients (22.5%) were discharged fully recovered (Figure 5). Death occurred in 15.4% of participants, often in the context of severe or recurrent hypoglycemia associated with complications such as CV events. Additionally, 9.7% of patients were discharged from the hospital with disabilities, patients reflecting impairments attributed to hypoglycemia-related complications. The outcomes of more than half of patients (52.4%) were unresolved at the end of the management period, often due to incomplete documentation.

### Association of demographic and clinical factors with hypoglycemia

In the binary logistic regression analysis evaluating the occurrence of hypoglycemia, several potential predictors were assessed, revealing varying associations (Table 6). Age groups did not significantly impact the likelihood of the occurrence of hypoglycemia, with odds ratios (OR) ranging from 0.600 (CI: 0.582–2.000, p > 0.05) to 1.480 (CI: 0.780–2.820, p > 0.05). Sex-based analysis, however, suggested a potential increased likelihood for males (OR = 2.088, CI: 0.131–3.106), indicating that they are twice more likely to experience hypoglycemia as compared to females, though it was not statistically significant (p = 0.078). Overall participant management, hypertension, and other comorbidities showed no significant association with hypoglycemia (OR = 0.963, CI: 0.708–1.310, p = 0.840; OR = 0.589, CI: 0.275–1.258, p = 0.250; OR = 0.622, CI: 0.362–1.063, p = 0.145). Other variables such as antidiabetic drugs (OR = 1.064, CI: 0.984–1.150, p = 0.188), previous CV conditions (OR = 1.575, CI: 0.163–15.265, p = 0.743), stroke (OR = 2.312, CI: 0.932–5.732, p = 0.129), renal disease (OR = 1.696, CI: 0.946–3.035, p = 0.139) showed a potential increase in likelihood, but lacked statistical significance. In contrast, insulin usage demonstrated a significant association (OR = 1.590, CI: 1.100–2.290, p = 0.010) with hypoglycemia, highlighting that it is a key factor affecting the likelihood of hypoglycemia.

**TABLE 6:** A binary logistic regression to identify key predictors of the occurrence of hypoglycemia.

Variable	Odd ratio	P-value	95% CI	
			Lower	Upper
Age groups (years)				
20–40 (Ref)				
41–50	1.480	0.293	0.780	2.820
51–60	1.090	0.630	0.874	1.890
61–70	0.750	0.310	0.377	1.420
71–80	0.750	0.430	0.385	1.310
81–90	0.600	0.180	0.582	2.000
Sex				
Female (Ref)				
Male	2.088	0.078	0.131	3.106
Antidiabetic drugs	1.064	0.188	0.984	1.150
Overall patient management	0.963	0.840	0.708	1.310
Previous CV condition	1.575	0.743	0.163	15.265
Stroke	2.312	0.129	0.932	5.732
Hypertension	0.589	0.250	0.275	1.258
Renal disease	1.696	0.139	0.946	3.035
Other comorbidities	0.622	0.145	0.362	1.063
Insulin	1.590	0.010	1.100	2.290

CI: confidence interval; CV: cardiovascular.

## Discussion

The present study provides a comprehensive insight into the demographic and clinical characteristics of T2DM patients who experienced hypoglycemia, as well as the associated management approaches and outcomes. The findings highlight some key aspects of hypoglycemia among this population, contributing to a better understanding of the risk factors and management strategies for hypoglycemia in T2DM patients.

The study revealed that hypoglycemic episodes were recorded in 41.9% of T2DM patients. The highest number of hypoglycemic episodes was 15 and occurred in 1.8% patients. Most of the patients (30, 27.0%) had two hypoglycemic episodes, followed by 26 (23.4%), 15 (13.5%), and 11 (9.9%) of patients who experienced one, three, and five episodes, respectively. The reported hypoglycemia episodes are similar to those reported by González-Vidal et al. [16] and higher than those reported in a study conducted by Miller CD et al. [17].

The demographic data demonstrated that the mean age of 58 years for patients aligns with the generally higher prevalence of T2DM among older adults. This finding is consistent with other studies which reported an increased risk of diabetes and its complications with advancing age. Accordingly, a study also observed a higher incidence of diabetes complications in older populations, highlighting the progressive nature of the disease with age [18]. Despite the prevalence of older adults in this study, the highest rates of hypoglycemia were observed in younger participants. This inverse relationship between T2DM age groups and hypoglycemia prevalence is notable, and it suggests that younger individuals with T2DM may be at a higher risk for hypoglycemic episodes. However, these findings may underestimate the true prevalence of hypoglycemia, as data were limited to available medical records from two tertiary hospitals. Mild episodes, particularly those not reported or documented, could have been missed, potentially influencing the observed associations with cardiovascular outcomes.

The decline in hypoglycemia rates with increasing age could be attributed to several factors. The observed inverse relationship between age and hypoglycemia prevalence may be attributed to a constellation of physiological, behavioral, and treatment-related factors that disproportionately affect younger individuals. It was shown that younger patients often exhibit heightened insulin sensitivity and are more likely to receive intensive glycemic control regimens, which increase their risk of hypoglycemic episodes. Additionally, their higher metabolic rate, lower glycogen reserves, and more variable dietary and activity patterns contribute to greater glucose fluctuations. These findings contrast with previous studies that suggested increased vulnerability among older adults due to polypharmacy, comorbidity and physiological changes in glucose metabolism and renal changes [19, 20]. However, the binary logistic regression analysis in this study did not find a statistically significant association between age and hypoglycemia, suggesting that while age may influence hypoglycemia rates, it is not an independent predictor when other factors are taken into account.

The sex distribution in the study was nearly equal. Interestingly, males exhibited a higher proportion of hypoglycemia compared to females. This sex disparity in hypoglycemia prevalence highlights potential differences in how males and females manage their diabetes or respond to treatment. In addition to the observed difference in hypoglycemia rates between sexes, the logistic regression analysis suggested a trend toward males being more likely to experience hypoglycemia. These findings are consistent with the higher rates of hypoglycemia observed in males [21]. The literature on sex differences in hypoglycemia has reported mixed findings, with some studies showing higher rates in males [21–23], aligning with the current study, while others have reported no significances [24, 25]. This suggests that sex-specific factors influencing hypoglycemia risk in T2DM require further investigation.

The study also examined the clinical characteristics of the study participants, focusing on CV conditions and comorbidities. A substantial proportion of patients had a history of CV conditions, and had had developed hypertension. The logistic regression analysis demonstrated an association between CV conditions and hypoglycemia, despite being non-statistically significant. A systematic review reported hypoglycemia as a strong predictor of CV diseases [26]. The presence of these comorbidities is significant, as they are known to complicate the management of diabetes and may increase the risk of hypoglycemia [27]. However, the logistic regression analysis did not find a significant association between these comorbidities and hypoglycemia. This suggests that while CV conditions and hypertension are prevalent in this population, they may not directly influence the occurrence of hypoglycemic episodes. Instead, the study found that insulin use was a significant predictor of hypoglycemia. This finding is consistent with previous research, which has identified insulin therapy as a key risk factor for hypoglycemia in patients with T2DM [16]. The significant association between insulin use and hypoglycemia underscores the importance of careful management and monitoring of insulin therapy to reduce the risk of hypoglycemic episodes in this population.

The study also explored the management strategies employed for patients who experienced hypoglycemia. Insulin was the most frequently used medication, followed by combinations of insulin with other antidiabetic drugs. The high use of insulin aligns with its role in managing blood glucose levels in T2DM patients, but also highlights the associated risk of hypoglycemia [17]. The study found that G50 was the most commonly used strategy to manage hypoglycemia. However, the fact that 25% of management approaches were not specified suggests a gap in documentation or standardized care practices, which could impact patient outcomes. In terms of overall management outcomes, the study found that 22.5% of patients achieved recovery, while 15.4% died, and 9.7% were discharged with disabilities. The finding that more than half of the patients (52.4%) were not reported cases indicates a lack of favorable clinical response to the available management strategies. These outcomes reflect the serious nature of hypoglycemia in T2DM and the need for effective management strategies to prevent adverse outcomes. The high mortality rate underscores the critical need for improved monitoring and individualized treatment plans for high-risk patients.

## Conclusion

This study highlights the complex interplay of demographic and clinical factors in the occurrence and management of hypoglycemia among T2DM. While younger age and males were associated with higher hypoglycemia rates, these factors were not significant predictors in the regression analysis, suggesting that other factors, particularly insulin use, play a more critical role in hypoglycemia risk. The findings emphasize the need for tailored management strategies, particularly regarding insulin therapy, to mitigate the risk of hypoglycemia and improve patient outcomes. Future studies should focus on further elucidating the factors contributing to hypoglycemia in different demographic groups and developing targeted interventions to address these risks.

## Limitations

The retrospective design of this study limits the ability to infer causality. Furthermore, important confounders such as diet, medication adherence, and comorbidities were not fully available in the dataset, which may have introduced bias in the estimated associations between hypoglycemia, CV outcomes, and mortality. Individual admission glucose levels, HbA1c values, and number of years of diabetes data were not consistently available in the medical records, limiting the precision of hypoglycemia assessment. Multivariate regression was not performed due to the limited number of events. Our study primarily aimed to provide descriptive data identify associations between hypoglycemia, cardiovascular outcomes, and mortality in patients with T2DM in Rwandan tertiary hospitals.

## Data Availability

The data analyzed in this study is subject to the following licenses/restrictions: The dataset contains confidential patient information collected and entered during routine clinical care and is restricted by institutional and ethical guidelines. It is not publicly available but may be accessed upon reasonable request with appropriate approval. Requests to access these datasets should be directed to JN, nbaptiste1988@gmail.com.
